# Supplementary material for: Modularized Genes in an Adrenal Pathway Reveal a Novel Mechanism in Hypertension Pathogenesis
Source: Int J Mol Sci. 2025 Apr 17;26(8):3782. doi: 10.3390/ijms26083782 (PMC12027864; doi:10.3390/ijms26083782)
Supplement: Supplementary file 1 [file ijms-26-03782-s001.zip › ijms-3474079-supplementary.pdf]

Supplemental Figure S1: Experimental designs in flow chart

Association/linkage of localizing QTLs by statistics (Already done in our previous work)

↓

Physiologically assessing blood pressure effect of a QTL (current studies onwards)

↓ ↓

Identifying a candidate gene for an individual QTL, *Cuedc1*

Analyzing combined effects of multiple QTLs

↓

Physiological characteristics of a QTL, *Cuedc1* ↔ A common pathway

↓

Identifying Cuedc1 pathway in mammalian common ancestors
and implicating interspecies translation of its physiological mechanisms
